# Management of tuberculosis by healthcare practitioners in Pakistan: A systematic review

**DOI:** 10.1371/journal.pone.0199413

**Published:** 2018-06-21

**Authors:** Christy A. Braham, Peter J. White, Nimalan Arinaminpathy

**Affiliations:** 1 MRC Centre for Outbreak Analysis & Modelling, Imperial College London, London, United Kingdom; 2 NIHR Health Protection Research Unit in Modelling Methodology, Imperial College London, London, UK; 3 Modelling & Economics Unit, National Infection Service, Public Health England, London, UK; Centre for the AIDS Programme of Research in South Africa (CAPRISA), SOUTH AFRICA

## Abstract

**Objective:**

To assess the quality of tuberculosis (TB) care in Pakistan, through determining comparison of healthcare practitioners’ knowledge and practices to national and international TB care guidelines.

**Methods:**

Studies reporting on knowledge, attitudes and practices of public and private practitioners with TB patients were selected through searching electronic databases and grey literature.

**Findings:**

Of 1458 reports, 20 full-texts were assessed, of which 11 met the eligibility and quality criteria; all studies focused on private sector care. Heterogeneity precluded meta-analysis. In 3 of 4 studies, over 50% of practitioners correctly identified a cough as the main TB symptom. However, 4 out of 6 studies showed practitioners’ compliance to be low (under 50%) for the use of sputum microscopy in diagnosis. The poorest quality care occurred in the later stages of treatment, with low compliance in prescribing practices for continuation-phase care and in monitoring and recording treatment progress, the latter of which is particularly critical for treatment success.

**Conclusion:**

TB care was variable and generally inadequate, with both a lack of knowledge and a small ‘know-do’ gap evident—practitioners did not use methods that they know they should use. A lack of recent evidence found suggests that the quality of current practices may not be fully captured and further research is needed, especially on non-allopathic, rural and public-sector contexts. Improved training of practitioners, greater availability of recommended diagnostic tools and expansion of public-private partnerships are suggestions for improving the quality of TB care in Pakistan.

## Background

Pakistan has the world’s fifth-highest tuberculosis (TB) prevalence, fourth-highest multi-drug resistant TB (MDR-TB) prevalence, and has 61% of the TB cases in the WHO Eastern Mediterranean Region [1]. TB was declared a public health emergency in Pakistan in 2001 and the National Treatment Program (NTP) initiative was relaunched with an expanded Directly Observed Therapy Short-course (DOTS) program [2]; this has achieved improvements in case detection and treatment success rates [3]. Despite these successes, around 70% of the population initially seek care from the private sector [4], in which implementing guidelines is challenging.

A previous scoping review of private practitioners (including all categories of TB care providers) involved in TB care in high-burden countries found that Pakistani practitioners showed low levels of knowledge and practice in several areas [5]. TB management in Pakistan has received less research attention than India, where a systematic review highlighted deficiencies in the quality of TB care, particularly amongst the private sector [6]. No such systematic review has been published on Pakistan. Our objective is to analyse all relevant data on the management of TB and the quality of care that patients receive in Pakistan, and to assess and identify where intervention is needed.

## Methods

No review protocol exists for this study.

### Search strategy

Medline, HMIC, EMBASE (via Healthcare Databases Advanced Search), Web of Science and Google Scholar were searched for the period 1995–2017, to capture studies conducted since the initial implementation of NTP with DOTS in Pakistan [7], with the most recent search completed on 9^th^ June 2017. Titles and abstracts were searched in English, using the terms “Pakistan” plus “tuberculosis knowledge”, “tuberculosis management” or “tuberculosis practice”, but also incorporating MeSH terms; full details are in **S1 Fig**. No restrictions on publication type were applied. Online journals were sought using the search terms, namely the Pakistan Journal of Medical Sciences and Pakistan Journal of Chest Medicine, as well as the gateway PakMediNet. Additionally, the reference lists of previously-retrieved papers were closely examined. Endnote X7.3 was used for storing references.

### Study eligibility

Practitioners in any health setting in Pakistan were included, including general practitioners, specialist physicians, nurses and non-clinical personnel. Allopathic and non-allopathic/traditional care contexts were also included; 70–80% of Pakistanis use non-Western, non-mainstream medicine such as Ayurveda, Homeopathy or Tibb-e-Unani [8]. Care of patients of any age and sex was included. Both pulmonary and extrapulmonary TB were included, as well as MDR-TB, XDR-TB and TB-HIV infections. Quantitative and qualitative studies of all study designs were considered.

Outcomes not reflecting the TB knowledge, attitudes or practices of practitioners in Pakistan were excluded. Studies containing outcome measures encompassing both public and private settings where data was not stratified by public/private context to enable effective comparison (in light of the importance of public and private sector relations for TB control), were also excluded.

### Quality and bias assessment

Bias was minimised in several ways. Location bias was minimised by searching Pakistan-based journals. Furthermore, thorough examination of all included literature allowed for identification of duplicated results across studies, thereby minimising duplication bias. However, as only literature in the English language was included, some language bias may exist.

Bias on an individual study level was assessed using an adapted version of the Newcastle-Ottawa Scale [9] to assess the quality of cross-sectional studies (see **S2 Fig**). The Scale contains three domains: Selection, Comparability and Outcome. Under Selection, a response rate was viewed as ‘satisfactory’ if it was at least 50%, while under Comparability a study needed to explain and control for confounding factors to be rated highly in this domain. Studies of inadequate quality (fewer than 5 stars out of ten) were excluded.

### Data extraction and analysis

Information on study characteristics and data on TB diagnosis and treatment were extracted (**Table 1**). Each outcome measure required data on practitioners’ knowledge or practices from at least three different studies, in order to qualify for analysis. When not provided in the papers, 95% confidence intervals were calculated for each item of data. Forest Plot Viewer was used to create forest plots for every outcome measure; each forest plot illustrated the ‘effect size’ (the item of data on practitioners’ knowledge or practices which has been extracted from the literature), as well as the sample size and confidence intervals.

**Table 1:** Summary of data extracted from literature.

Author (year)	Location (province/territory)	Setting	Provider	Medical practice	Study population (response rate)	Data collection method	Sampling method	ISTC Standards measured (see Table 2)
Ahmed et al. (2009) [20]	Taluka Thatta (Sindh)	Rural	Private	Allopathic	22 primary care doctors (44%)	Questionnaire	Convenience	1, 2, 8, 10, 13
Arif et al. (1998) [21]	Karachi (Sindh)	Urban	Private	Allopathic	229 patients	Questionnaire & patient records	Random	2, 8, 10
Fatima et al. (2014) [22]	Rawalpindi, Khushab, Lodhran & Rajanpur (Punjab); Larkana & Mirpurkhas (Sindh); Swat, Buner & Battgram (Khyber-Pakhtunkhwa); Zhob, Lasbella & Washuk (Balochistan)	Urban & Rural	Private	Allopathic	1700 practitioners incl. GPs, hospital physicians & medical assistants (90%)	Patient records	Random	2
Hussain et al. (2005) [23]	Rawalpindi (Punjab)	Urban	Private	Allopathic	53 GPs, specialists & other doctors	Prescription records & standardised patient	Random	8
Khan et al. (2003) [24]	Karachi (Sindh)	Urban	Private	Allopathic	120 GPs (85.1%)	Questionnaire	All in population included	1, 2, 8, 10, 13
Khan et al. (2005) [25]	Karachi (Sindh)	Urban	Private	Allopathic	120 general practitioners	Questionnaire	All in population included	2, 8, 10, 13
Khan & Hussain (2003) [27]	Karachi (Sindh)	Urban	Private	Allopathic	362 patients	Patient & pharmacy records	Random	8
Marsh et al. (1996) [28]	Karachi & Hyderabad (Sindh)	Urban	Private	Allopathic & non-allopathic	68 general practitioners & 152 patients	Questionnaire	Non-random	2, 8, 10
Rizvi & Hussain (2001) [29]	Karachi (Sindh)	Urban	Private	Allopathic	150 general practitioners	Questionnaire	Random	1, 2, 8, 10
Shah et al. (2003) [30]	Lahore & Rawalpindi (Punjab)	Urban	Private	Allopathic	245 doctors	Questionnaire	Random	1, 2, 8, 10, 13
Shehzadi et al. (2005) [31]	Gilgit, Skardu & Hunza (Gilgit Baltistan), Haripur, Peshawar & Abbotabad (Khyber-Pakhtunkhwa)	Urban & Rural	Private	Allopathic	88 general practitioners	Questionnaire & pharmacy records	Convenience	2, 10

Characteristics of 11 studies on knowledge and practices in relation to TB care which were included in systematic review, following quality assessment

Results for each identified measure were compared with the National Guidelines for the Control of Tuberculosis in Pakistan [10], and the International Standards of Tuberculosis Care (ISTC) [11]. The second edition of the ISTC was used instead of the third edition (2014) due to the latter’s emphasis on using Xpert MTB/RIF for diagnosis, as this tool is not yet widely accessible in Pakistan. The ISTC was included as a benchmark for a ‘good’ standard of care in order to assess the quality of TB care in Pakistan; in this way, our systematic review does not seek to formally measure compliance with the ISTC standards, as some literature may predate these standards. **Table 2** shows example comparisons of Pakistan’s national guidelines with the ISTC.

**Table 2:** National and international guidelines on TB care.

ISTC Standards (Tuberculosis Coalition for Technical Assistance)	National Guidelines for the Control of Tuberculosis in Pakistan (NTP)
ISTC Standard 1: All persons with otherwise unexplained productive cough lasting 2–3 weeks or more should be evaluated for TB.	The most common symptom of TB is a productive cough for more than 2 weeks, which may be accompanied by other respiratory/constitutional symptoms
ISTC Standard 2: All patients who are capable of producing sputum suspected of having pulmonary TB should have at least 2 sputum specimens submitted. When possible, at least one early morning specimen should be obtained.	All adult patients suspected of having pulmonary TB should have at least two sputum specimens examined for AFB smear microscopy in a quality-assured laboratory
ISTC Standard 8: All patients (including those with HIV infection) who have not been treated previously should receive an internationally accepted first-line treatment regimen using drugs: The initial phase should consist of two months of isoniazid (INH), rifampicin (RIF), pyrazinamide (PZA), and ethambutol (EMB). The continuation phase should consist of isoniazid and rifampicin given for four months. Fixed dose combinations of 2,3 or 4 drugs are highly recommended.	During the initial intensive phase four drugs (Isoniazid, Rifampicin, Pyrazinamide and Ethambutol “HRZE”) are administered under observation daily for a period of two months (sixty doses). During the continuation phase, isoniazid and rifampicin (HR) are administered daily for four months. Fixed dose combinations with proven bio-availability are preferred over individual drugs preparations.
ISTC Standard 10: Response to therapy in patients with pulmonary tuberculosis should be monitored by follow-up sputum microscopy (two specimens) upon completion of the initial phase of treatment (two months). If the sputum smear is positive, they should be examined again at 3 months and, if still positive, culture and drug susceptibility testing should be performed. In patients with extrapulmonary TB and in children, the response to treatment is best assessed clinically.	Sputum smear is done at the end of 2 months, if smear is negative, the continuation phase will start. However if sputum smear is positive, a repeat test will be carried out.
ISTC Standard 13: A written record of all medications given, bacteriologic response, and adverse reactions should be maintained for all patients.	Records of treatment must be kept. The care-giver must check the regularity of drug intake. Treatment outcomes must be assigned to every patient.

Comparisons of Pakistan’s national TB guidelines (2015) with the International Standards for Tuberculosis Care [ISTC] (2009)

## Results

### Study selection

**Fig 1:**
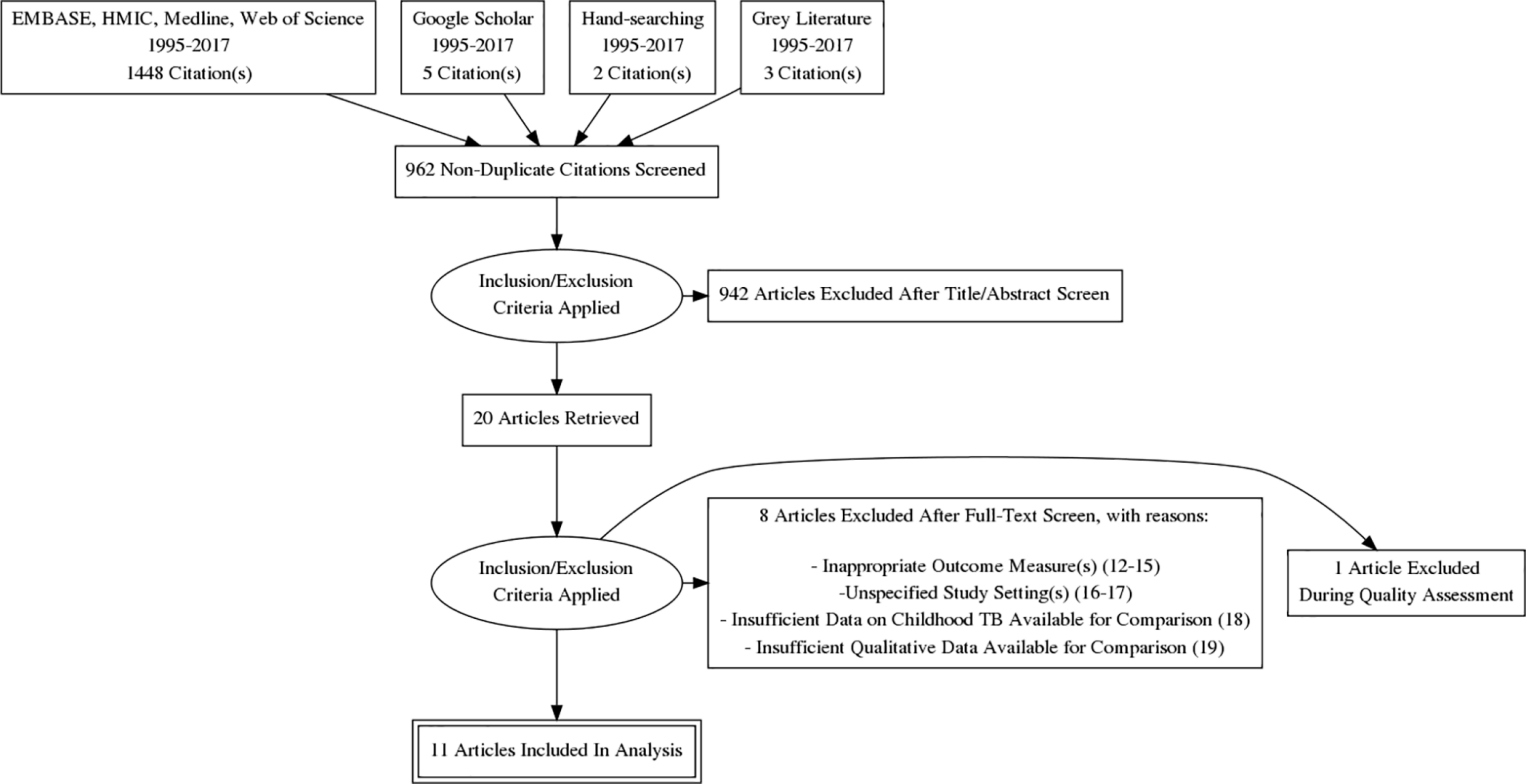
PRISMA flow chart. PRISMA flow chart outlining the search procedure for selecting studies on knowledge and practices in relation to TB care for the systematic review.

According to the PRISMA flow chart in **Fig 1**, 1448 citations (titles and abstracts) were retrieved through searching electronic databases. Additionally, five citations were found using Google Scholar, three citations through backwards reference searching and two citations through the perusal of Pakistan-based journals, bringing the number of citations retrieved using the search strategy to 1458. 962 of these citations were identified as being non-duplicates, leading to the exclusion of 942 citations after title and abstract screening. Twenty full-text articles were identified as being of direct relevance to the research question [12–31]. Eight articles did not meet the eligibility criteria [12–19], with 12 articles deemed eligible in the first instance [20–31].

### Results of quality & bias assessment

The 12 studies meeting the eligibility criteria scored between 4 and 7 stars out of 10 overall on the adapted Newcastle-Ottawa Scale, indicating mediocre to moderate quality of research. One study was excluded due to lower quality, having scored four stars [26]. A full summary of the results of the quality assessment can be seen in **S1 Table**.

### Study characteristics

One study was excluded for poor quality (details in **S1 Table**). The remaining eleven studies covered all 4 of Pakistan’s provinces (Sindh, Punjab, Balochistan and Khyber-Pakhtunkhwa) and one disputed territory (Gilgit-Baltistan) [**Table 1**]. Urban contexts were overwhelmingly common. Ten studies focused purely on allopathic settings, while non-allopathic/traditional medicine was represented in 1 study. No public sector contexts were represented. All studies focused on adult pulmonary TB and no data was provided on extrapulmonary TB, MDR-TB, XDR-TB, TB-HIV or any TB infection in children. All were quantitative and cross-sectional, with questionnaires, patient records, pharmacy / prescription records and standardised patients used for data collection.

### Outcome measures

Data on knowledge and practices were extracted from eleven studies and compared to the national guidelines (**Table 2**). Eleven outcome measures each contained data from at least 3 sources; eight are described here, while the remaining three are in **S1 Other Outcome Measures**. Of these outcome measures, eight offered direct relevance to several of the ISTC ‘Standards’, while the remaining three were not covered by the ISTC; of these three outcome measures, one was referred to in the national guidelines and two were included in this research as additional observations. ‘Knowledge’ and ‘practice’ of guidelines were distinguished wherever possible. Heterogeneity precluded formal meta-analysis.

#### ISTC Standard 1: Knowledge of an unexplained cough as a main symptom of tuberculosis

**Fig 2:**
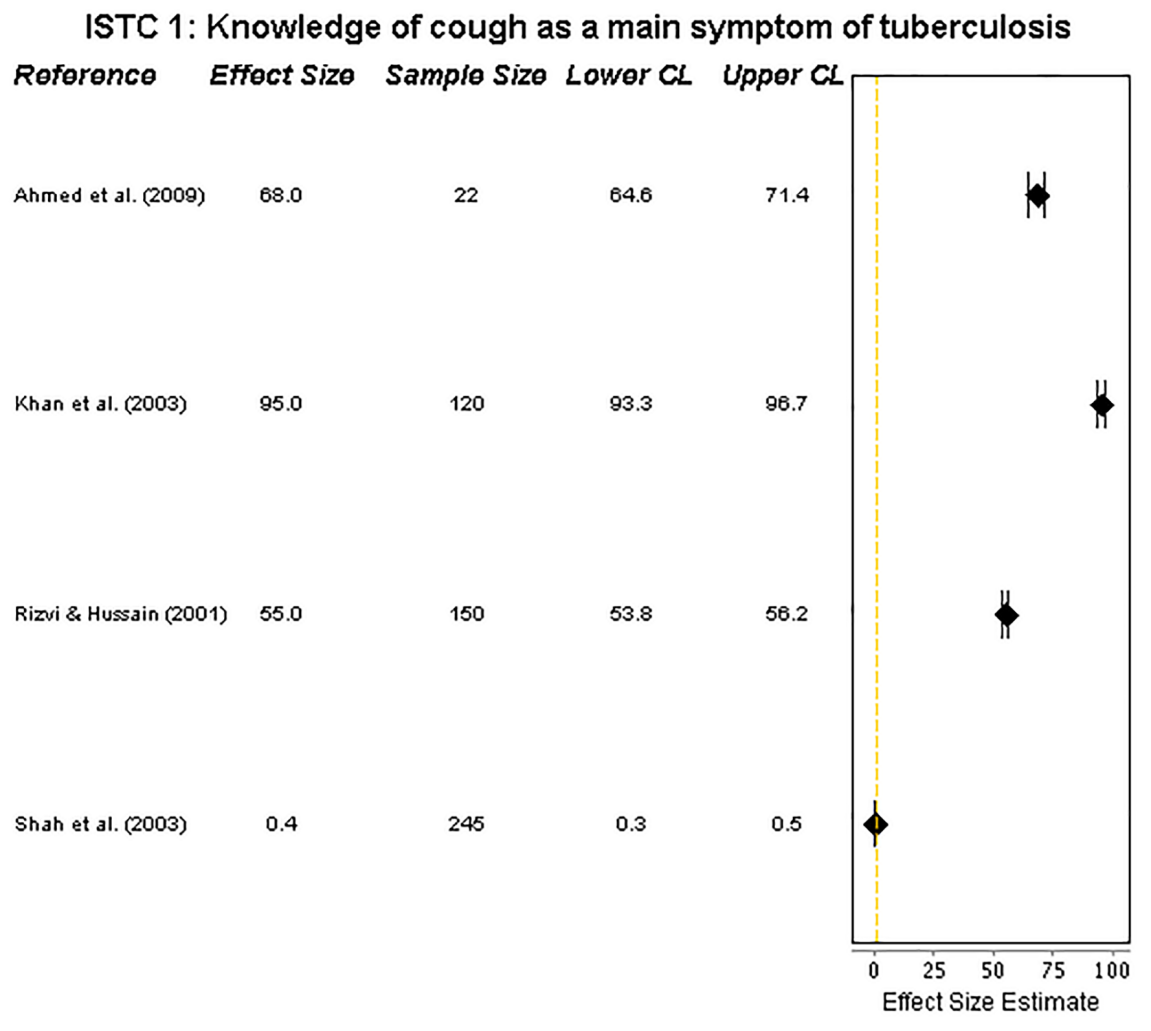
Forest plot on cough. Forest plot visualising data on practitioners’ knowledge of cough as a main symptom of tuberculosis (ISTC Standard 1). CL = 95% Confidence Level.

Four studies provided data on practitioners’ knowledge of cough as the principal symptom of pulmonary TB. All but one [29] reported that more than half of the practitioners were aware of the significance of a cough as a symptom. There was, however, very wide heterogeneity, ranging from 0.4% [29] to 95% [23] (**Fig 2)**.

#### ISTC Standard 2: Diagnosing pulmonary tuberculosis using sputum microscopy

**Fig 3:**
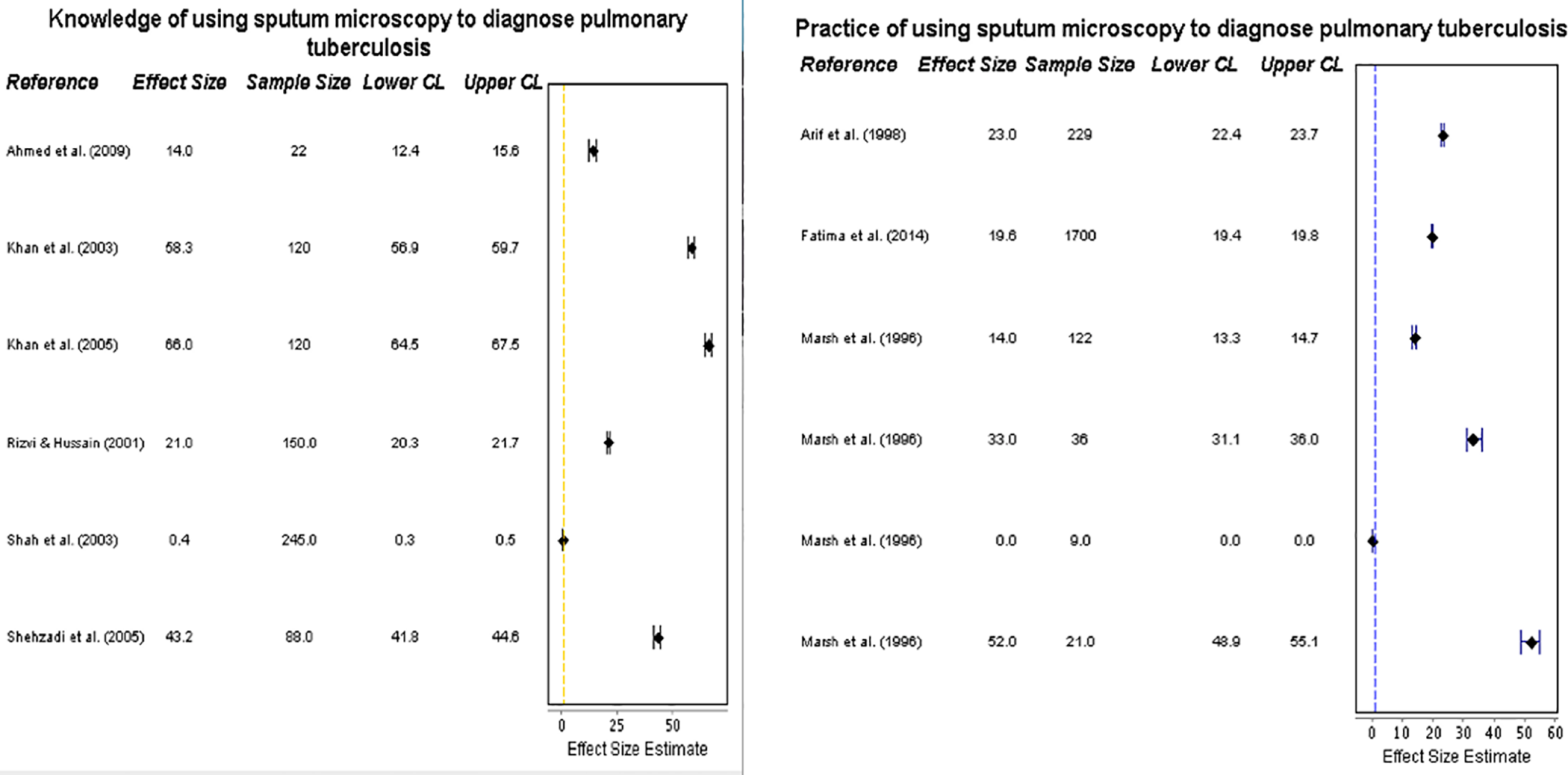
Forest plots on diagnosis knowledge and practice. **(a)** Forest plot visualising data on practitioners’ knowledge of using sputum microscopy for the diagnosis of pulmonary tuberculosis (ISTC Standard 2). CL = 95% Confidence Level. **(b)** Forest plot visualising data on practitioners’ practice of using sputum microscopy for the diagnosis of pulmonary tuberculosis (ISTC Standard 2). Marsh et al. (1996) includes 4 sets of data for this ISTC Standard, reflecting 4 different practitioner groups. CL = 95% Confidence Level.

Practitioners’ knowledge of using sputum microscopy for diagnosing the disease, and their actual practice of it, are compared in the forest plots in **Fig 3A and Fig 3B**. Six studies assessed practitioners’ *knowledge*, which ranged from 14% [20] to 66% [24]. A poor level of knowledge was seen, with 4 studies reporting < 50% and 3 reporting <30% of the practitioners knowing the importance of sputum microscopy as the sole tool needed for TB diagnosis. There were 6 sources of data on *practice*; use of sputum microscopy was heterogeneous and low, ranging from 0% [28] to 52% [28] (both data items in this range were extracted from the same study) with 5 studies reporting less than 50%, and four reporting <30%.

#### ISTC Standard 8: Practice of prescribing the recommended drug regimens and prescribing in fixed-dose combinations

**Fig 4:**
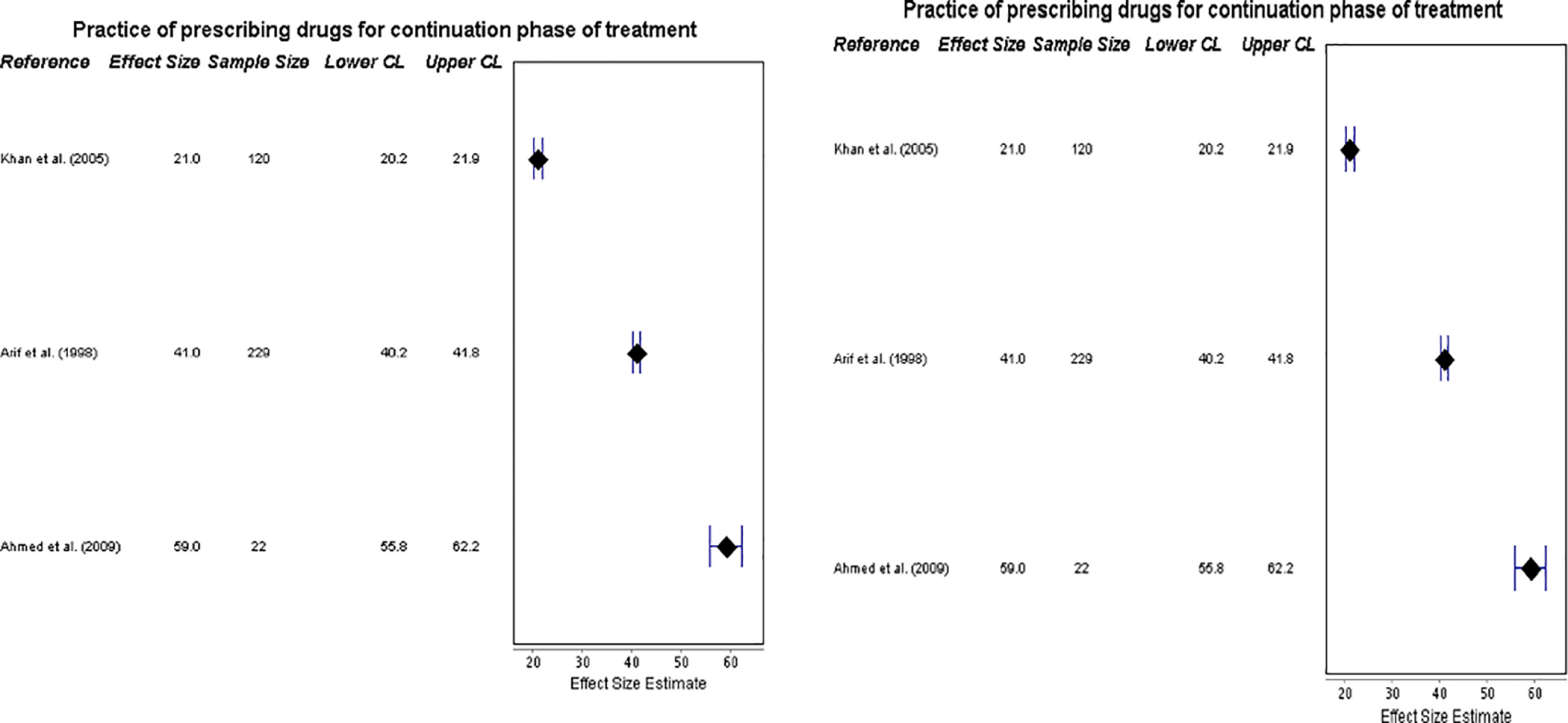
Forest plots on prescriptions for intensive and continuation phases. **(a)** Forest plot visualizing data on practitioners’ compliance with prescribing drugs for the intensive phase of TB treatment (ISTC Standard 8). CL = 95% Confidence Level. **(b)** Forest plot visualizing data on practitioners’ compliance with prescribing drugs for the continuation phase of TB treatment (ISTC Standard 8). CL = 95% Confidence Level.

Sufficient data was available to provide three outcome measures (on prescribing for the intensive and continuation phases, and in fixed-doses) which were all of direct relevance to this Standard, though there was an insufficient amount of data on the range of doses and durations of the drugs prescribed and ‘knowledge’ and ‘practice’ could not be distinguished here. Six sources provided insight into prescribing the correct drugs for the initial intensive phase of treatment, for new patients of TB. Results ranged from 23% [29] to 83% [25]; apart from one study [29], all others reported more than 73% practitioners meeting this standard of care. There was relatively wide heterogeneity (**Fig 4A**), indicating reasonable quality of care.

However, the three studies which provided data on correct prescribing of drugs for new patients during the subsequent continuation phase of treatment showed less promising results. Though less heterogeneous, and ranging from 21% [25] to 59% [20], this is suggestive of poor standards of care (also **Fig 4B).**

**Fig 5:**
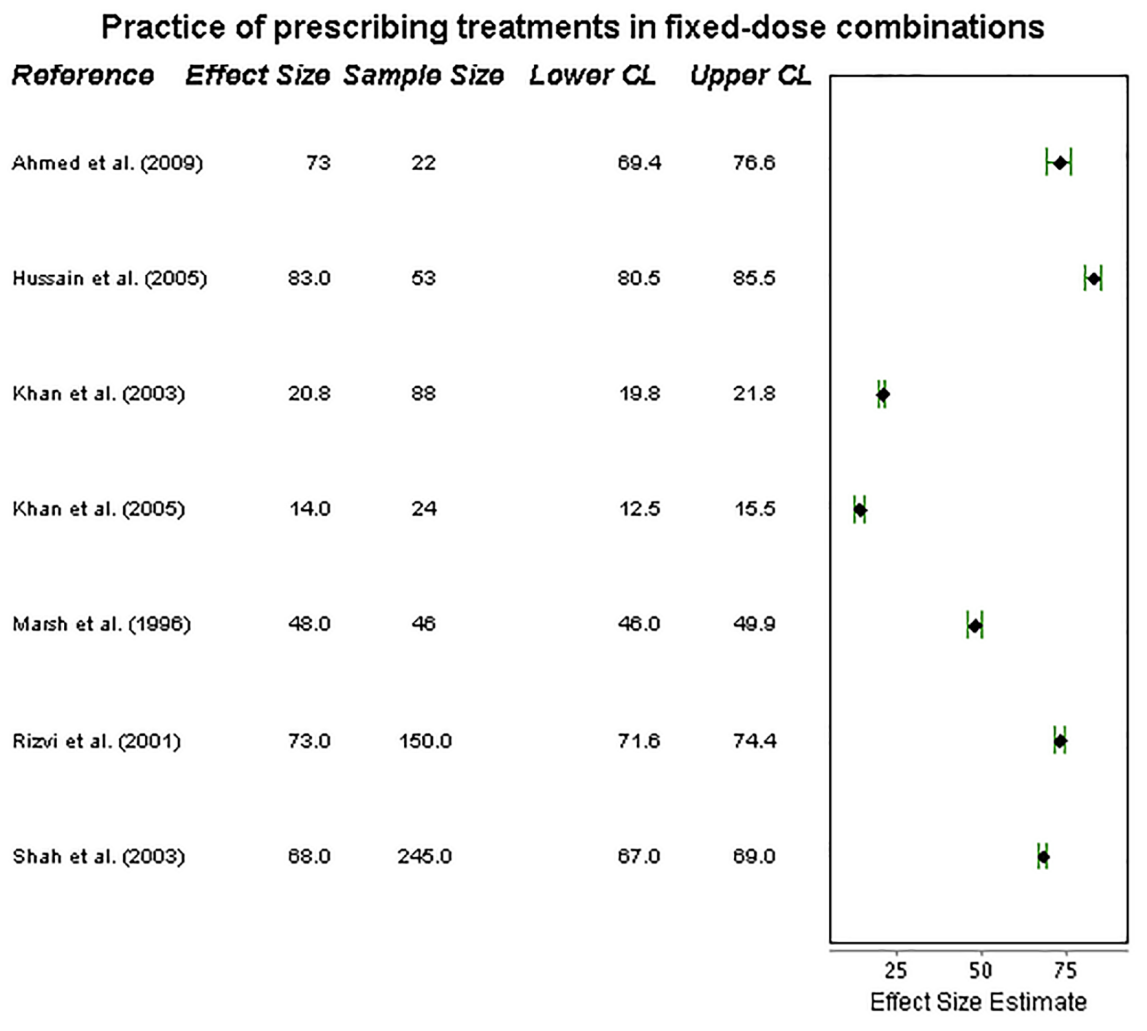
Forest plot on prescribing in fixed doses. Forest plot visualizing data on practitioners’ compliance with prescribing treatments in fixed-dose combinations (ISTC Standard 8). CL = 95% Confidence Level.

Finally, the practice of prescribing fixed-dose drug regimens was examined in seven studies. Wide heterogeneity was seen (**Fig 5**), with results ranging from 14% [25] to 83% [23] of practitioners prescribing treatments in this way. Four studies suggested that a minimum of around 70% of practitioners prescribed using fixed-dose regimens, although the remaining three studies saw practice of this fall below 50%. This implies a moderate standard of care.

#### ISTC Standard 10—knowledge of using sputum microscopy to assess clinical progress after the initial phase of treatment

**Fig 6:**
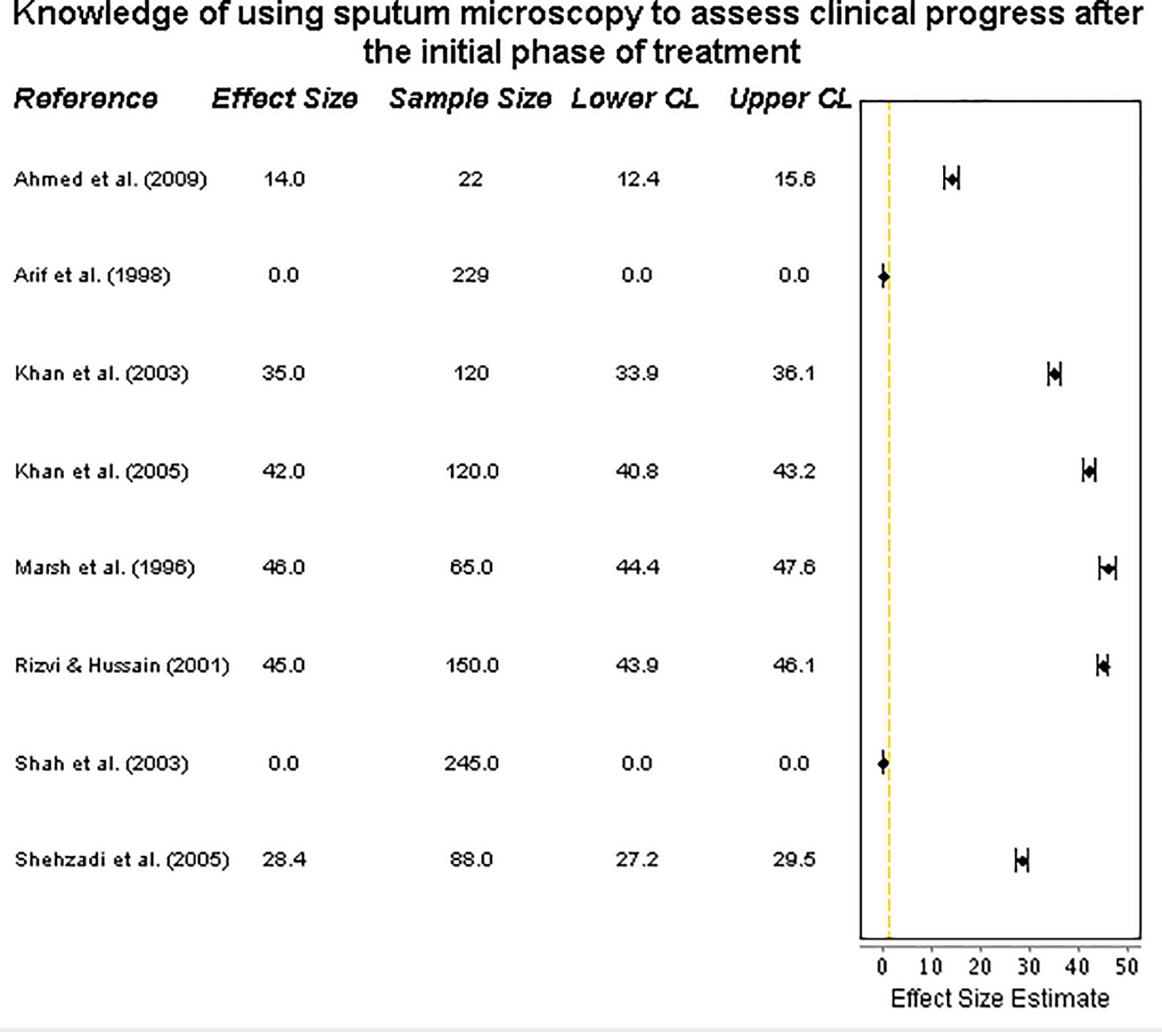
Forest plot on assessing clinical progress. Forest plot visualising data on practitioners’ knowledge of using sputum microscopy to monitor treatment progress (ISTC Standard 10). CL = 95% Confidence Level.

Data was extracted from eight studies. Distinguishing between ‘knowledge’ and ‘practice’ was not possible for this outcome, and the *number* of sputum specimens collected per patient could not be described. The proportion of practitioners using sputum microscopy for this purpose was very low (**Fig 6**), ranged from 0% [21,30] to 46% [28], with four studies reporting <30%. In all studies, less than half of practitioners knew to track their patients’ progress over the course of treatment using this recommended diagnostic tool.

#### ISTC Standard 13—practice of recording treatments and their outcomes

**Fig 7:**
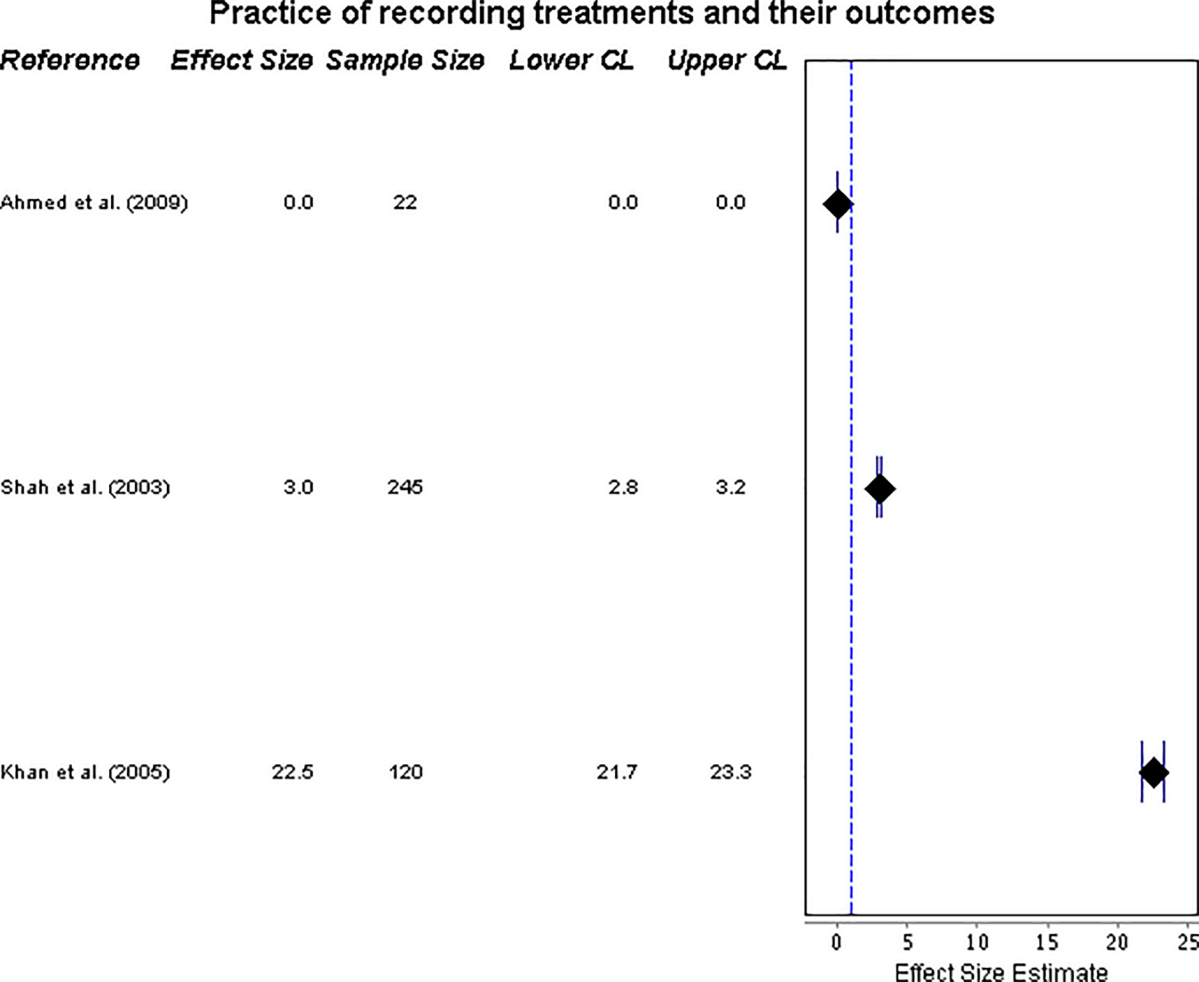
Forest plot on recording treatments. Forest plot visualising data on practitioners’ practice of recording treatments and their outcomes (ISTC Standard 13). CL = 95% Confidence Level.

Provision of records of treatment was assessed by three studies, and was very low, ranging from 0% [20] to 22.5% [24] (see forest plot, in **Fig 7**).

#### Comparison across study contexts

A comparison of results was not possible between public and private contexts, owing to the fact that all included studies were found to be based solely on private practitioners (in particular, medical doctors). A very small amount of data regarding non-allopathic/traditional medicine suggested that such practitioners do not attempt to use sputum microscopy for the diagnosis of TB (ISTC Standard 2), and that they the lowest level of adherence to this guideline, 0% [29].

Data was included from rural as well as urban contexts. While two studies included samples of practitioners drawn from a range of urban and rural areas with no difference observed between practitioners’ knowledge or practices based on urban or rural context [31,22], the single study focussing exclusively on a rural context followed the same trends previously identified in the outcome measures—that TB care was generally inadequate [20]. Notably, in comparison with urban contexts, this rural study showed the highest standard of drug regimen prescribing for continuation phase treatment (59%, ISTC Standard 8) and the lowest standard of recording treatments (0%, ISTC Standard 13).

## Discussion

The private healthcare sector in Pakistan poses both challenges and opportunities for the control of TB in the country: for example, recent, innovative initiatives to engage with this sector have shown notable successes in promoting TB case detection [32]. In this context, the present work–to our knowledge the first systematic review of TB care in Pakistan–confirms that standards of care have been variable and typically inadequate, ranging from *very low* (protocols correctly followed by under 30% of practitioners) to *moderate* (protocols correctly followed by between 50–70% practitioners).

Knowledge of the protocol for sputum microscopy was notably poor and its use in practice was even poorer. This suggests a ‘know-do’ gap, as found in India [6]; there is some difference between what practitioners know and what they do in reality. Many practitioners did not use smear microscopy as a method of diagnosis. Where practitioners did use smear microscopy, it was not used as the sole method of diagnosis, instead being accompanied by other tools, chiefly chest X-ray [20,22,29,30]. Whilst chest X-ray can assist diagnosis of sputum-negative pulmonary TB [26], the practitioners observed often used chest X-rays before any sign of sputum-negative disease was noted. Widespread use of alternative tests instead of sputum microscopy may be motivated by practitioners’ financial considerations, with X-ray services often commanding higher fees [25].

Practitioners generally had a moderate level of knowledge of an unexplained cough as the principal symptom of pulmonary TB, but many practitioners showed poor knowledge of the diversity of symptoms. Differing levels of awareness of the latter suggests that TB cases not reporting an unexplained cough may be missed.

Regarding treatment, while a reasonable number of practitioners prescribed drug regimens in fixed-dose combinations, lower standards were seen when treating patients in the continuation phase than in the initial intensive phase. Additionally, there was particularly low awareness of the importance of sputum microscopy for measuring treatment progress; low use (<40%) of sputum microscopy for treatment monitoring has also been reported in India [6]. Failure to produce treatment records was common, similar to previous research in other TB high-burden countries [5,6]. Poor monitoring and recording of care is detrimental for TB control [33], contributing towards low levels of TB treatment success, including patient loss to follow-up and treatment failure. This is critical in that it promotes antibiotic resistance, therefore hampering the success of TB control initiatives.

This study identified a number of limitations of published research. Despite the use of broad inclusion criteria to capture literature representing the diversity of practitioners in the public and private sectors in Pakistan, *allopathic*, *medically-qualified and urban-based private practitioners* formed the overwhelming majority of the studies. Most notably, public practitioners were not represented, though inclusion criteria sought to include them, meaning that comparison between public and private contexts was not possible [6]. One study was excluded because its public/private setting could not be ascertained and therefore could have introduced bias, yet it reported practitioners’ TB knowledge to be of a good standard [16]. While all included studies focused on practitioners providing care in the private sector, it is not known if these same practitioners simultaneously worked in the public sector. In addition to this, only studies of pulmonary TB were found. With the search being conducted in English only, studies of relevance published in Urdu may have been missed. Although reporting bias was minimised during the literature search, bias may have contributed to heterogeneity across outcome measures, perhaps due to false reporting of practitioners’ behaviours and missing data (through abstraction from patient or pharmacy records). Finally, there was a lack of studies published in recent years—with a number of the included studies published more than a decade ago—which allows for the possibility that the standards of TB care could have changed.

Further research is required into treatment practices, including the drug dosages being prescribed by practitioners. Investigation of the effect of poor management on patient compliance and loss to follow-up may add useful context, as suggested previously [21]. There is substantial need for research into diagnosis and management of non-pulmonary TB, paediatric TB, MDR-TB and XDR-TB. Additionally, future studies should formally measure practitioners’ compliance against each of the most recently-published ISTC standards, to provide the best assessment of the quality of care and to show how the quality of TB care in Pakistan may have improved. Finally, the quality of any subsequent research into the management of TB in Pakistan would be limited without greater focus on non-allopathic/traditional practitioners and those based in rural areas—who many patients preferentially engage with [34].

## Conclusion

TB management in Pakistan requires improvement in all areas identified in this research, in relation to diagnosis, treatment, and monitoring. An obvious intervention would be improved education of practitioners on guidelines with continuous assessment and monitoring. This must be offered to the wide variety of private practitioners with TB patients. Empowerment of private practitioners through better education and increased engagement is likely to affect the quality of care that they provide; previous work in the country has shown the potential value of the private sector in identifying TB cases in the community [32]. Use of only appropriate diagnostic tools needs to be encouraged, in order to address the “know-do” gap. This should include increasing access to appropriate diagnostic technology (including rapid tests such as Xpert MTB/RIF), and addressing incentives to use inappropriate tests. Additionally, continued expansion of Public-Private partnerships could lead to better management of TB patients, particularly through treatment success. The aforementioned activities would help to combat the TB epidemic in Pakistan and aid the search for the ‘missing 3 million’ individuals worldwide who are left untreated of TB [35].

## Supporting information

S1 PRISMA 2009 Checklist PRISMA 2009 checklist describing essential components of the systematic review. (PDF)

S1 Fig Full electronic database search input. Full search input for EMBASE, HMIC and Medline, conducted via Healthcare Databases Advanced Search (HDAS) on 9th June 2017. Search terms were also used within the Web Of Science database directly.
(PDF)

S2 Fig Adapted Newcastle-Ottawa Scale. Adapted Newcastle-Ottawa Scale for cross-sectional studies, devised by Herzog et al. [9] used as a quality assessment tool for studies identified as meeting the eligibility criteria.
(PDF)

S3 Fig Forest plot on range of symptoms. Forest plot visualising data on practitioners’ knowledge of the range of symptoms of tuberculosis. CL = 95% Confidence Level.
(TIF)

S4 Fig Forest plot on diagnosing and not referring. Forest plot visualizing data on practitioners’ practice of diagnosing patients and not referring them onto other practitioners. CL = 95% Confidence Level.
(TIF)

S5 Fig Forest plot on treating and not referring. Forest plot visualizing data on practitioners’ practice of treating patients and not referring them onto other practitioners. CL = 95% Confidence Level.
(TIF)

S1 Table Quality assessment. Outcome of the quality assessment of studies identified from the literature search and meeting the eligibility criteria, using the Newcastle-Ottawa Scale.
(DOCX)

S1 Other Outcome Measures Further information on non-ISTC outcomes measured as part of the review. (DOCX)
