# Supplementary material for: Management of tuberculosis by healthcare practitioners in Pakistan: A systematic review
Source: PLoS One. 2018 Jun 21;13(6):e0199413. doi: 10.1371/journal.pone.0199413 (PMC6013248; doi:10.1371/journal.pone.0199413)
Supplement: S1 Fig — Full search input for EMBASE, HMIC and Medline, conducted via Healthcare Databases Advanced Search (HDAS) on 9th June 2017. Search terms were also used within the Web Of Science database directly. (PDF) [file pone.0199413.s002.pdf]

1. EMBASE; PAKISTAN/ OR PAKISTANI/; 18839 results

2. EMBASE; ABDOMINAL TUBERCULOSIS/ OR CENTRAL NERVOUS SYSTEM TUBERCULOSIS/ OR CONGENITAL TUBERCULOSIS/ OR DRUG RESISTANT TUBERCULOSIS/ OR EXTENSIVELY DRUG RESISTANT TUBERCULOSIS/ OR EXTRAPULMONARY TUBERCULOSIS/ OR INTESTINE TUBERCULOSIS/ OR KIDNEY TUBERCULOSIS/ OR LARYNGEAL TUBERCULOSIS/ OR LATENT TUBERCULOSIS/ OR LUNG TUBERCULOSIS/ OR MILIARY TUBERCULOSIS/ OR MULTIDRUG RESISTANT TUBERCULOSIS/ OR MYCOBACTERIUM TUBERCULOSIS/ OR OCULAR TUBERCULOSIS/ OR POSTPRIMARY TUBERCULOSIS/ OR PRIMARY TUBERCULOSIS/ OR SKIN TUBERCULOSIS/ OR TUBERCULOSIS/ OR UROGENITAL TUBERCULOSIS/; 205026 results

3. EMBASE; TB.ti,ab; 43107 results

4. EMBASE; SHORT COURSE THERAPY/; 9543 results

5. EMBASE; DOTS.ti,ab; 16299 results

6. EMBASE; CASE MANAGEMENT/ OR DISEASE MANAGEMENT/ OR HEALTH CARE MANAGEMENT/ OR HOSPITAL MANAGEMENT/ OR KNOWLEDGE MANAGEMENT/ OR MANAGEMENT/ OR ORGANIZATION AND MANAGEMENT/; 481857 results

7. EMBASE; CLINICAL PRACTICE/ OR EVIDENCE BASED PRACTICE/ OR GENERAL PRACTICE/ OR HEALTH CARE PRACTICE/ OR MEDICAL PRACTICE/ OR NURSING PRACTICE/ OR PRACTICE GUIDELINE/ OR PROFESSIONAL PRACTICE/; 647519 results

8. EMBASE; KNOWLEDGE/ OR KNOWLEDGE BASE/ OR KNOWLEDGE MANAGEMENT/ OR NURSING

KNOWLEDGE/ OR PROFESSIONAL KNOWLEDGE/; 40867 results

9. EMBASE; ATTITUDE/ OR ATTITUDE TO HEALTH/ OR ATTITUDE TO ILLNESS/ OR EMPLOYEE ATTITUDE/ OR HEALTH PERSONNEL ATTITUDE/ OR PHYSICIAN ATTITUDE/; 243571 results

10. EMBASE; COMPLIANCE/ OR COMPLIANCE TO PROTOCOL/ OR COMPLIANCE TO THERAPY/ OR COMPLIANCE TO TREATMENT/; 116925 results

11. EMBASE; 2 OR 3 OR 4 OR 5; 240469 results

12. EMBASE; 6 OR 7 OR 8 OR 9 OR 10; 1392209 results

13. EMBASE; 1 AND 11 AND 12; 120 results

14. EMBASE, HMIC, Medline; 1 AND 11 AND 12; 124 results
