# Supplementary material for: Management of tuberculosis by healthcare practitioners in Pakistan: A systematic review
Source: PLoS One. 2018 Jun 21;13(6):e0199413. doi: 10.1371/journal.pone.0199413 (PMC6013248; doi:10.1371/journal.pone.0199413)
Supplement: S2 Fig — Adapted Newcastle-Ottawa Scale for cross-sectional studies, devised by Herzog et al. [9] used as a quality assessment tool for studies identified as meeting the eligibility criteria. (PDF) [file pone.0199413.s003.pdf]

Newcastle-Ottawa Scale adapted for cross-sectional studies

Selection: (Maximum 5 stars)

1) Representativeness of the sample:
   - a) Truly representative of the average in the target population. * (all subjects or random sampling)
   - b) Somewhat representative of the average in the target population. * (non-random sampling)
   - c) Selected group of users.
   - d) No description of the sampling strategy.

2) Sample size:
   - a) Justified and satisfactory. *
   - b) Not justified.

3) Non-respondents:
   - a) Comparability between respondents and non-respondents characteristics is established, and the response rate is satisfactory. *
   - b) The response rate is unsatisfactory, or the comparability between respondents and non-respondents is unsatisfactory.
   - c) No description of the response rate or the characteristics of the responders and the non-responders.

4) Ascertainment of the exposure (risk factor):
   - a) Validated measurement tool. **
   - b) Non-validated measurement tool, but the tool is available or described.*
   - c) No description of the measurement tool.

Comparability: (Maximum 2 stars)

1) The subjects in different outcome groups are comparable, based on the study design or analysis. Confounding factors are controlled.
   - a) The study controls for the most important factor (select one). *
   - b) The study control for any additional factor. *

Outcome: (Maximum 3 stars)

1) Assessment of the outcome:
   - a) Independent blind assessment. **
   - b) Record linkage. **
   - c) Self report. *
   - d) No description.

2) Statistical test:
   - a) The statistical test used to analyze the data is clearly described and appropriate, and the measurement of the association is presented, including confidence intervals and the probability level (p value). *
   - b) The statistical test is not appropriate, not described or incomplete.
