# Supplementary figures and images for: Management of tuberculosis by healthcare practitioners in Pakistan: A systematic review
Source: PLoS One. 2018 Jun 21;13(6):e0199413. doi: 10.1371/journal.pone.0199413 (PMC6013248; doi:10.1371/journal.pone.0199413)

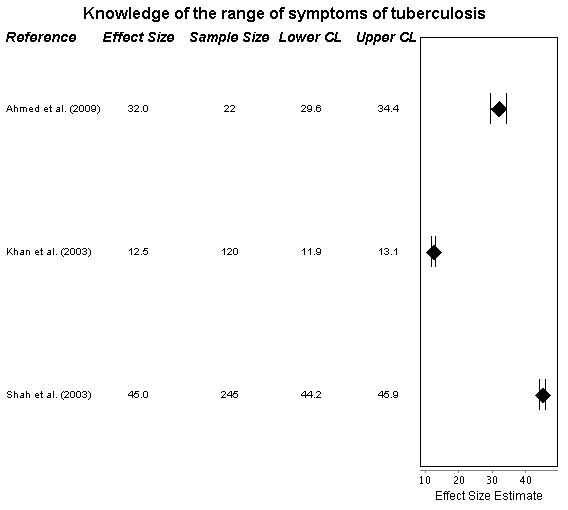

Supplement: S3 Fig — Forest plot visualising data on practitioners’ knowledge of the range of symptoms of tuberculosis. CL = 95% Confidence Level. (TIF) [file pone.0199413.s004.tif]

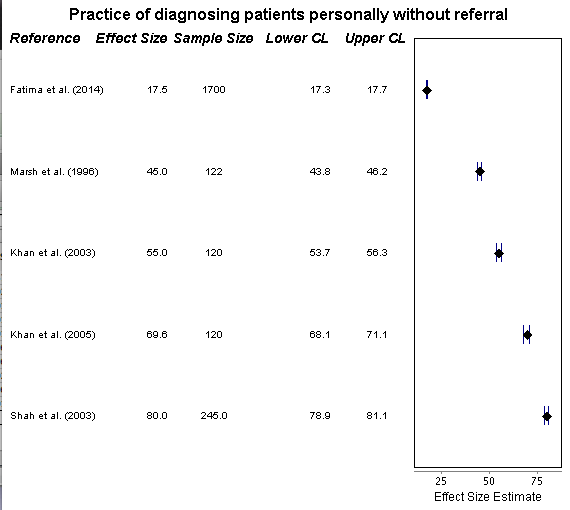

Supplement: S4 Fig — Forest plot visualizing data on practitioners’ practice of diagnosing patients and not referring them onto other practitioners. CL = 95% Confidence Level. (TIF) [file pone.0199413.s005.tif]

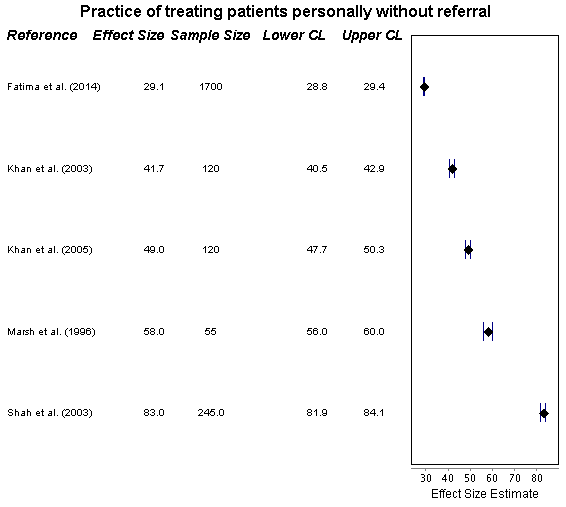

Supplement: S5 Fig — Forest plot visualizing data on practitioners’ practice of treating patients and not referring them onto other practitioners. CL = 95% Confidence Level. (TIF) [file pone.0199413.s006.tif]
