# Supplementary material for: Management of tuberculosis by healthcare practitioners in Pakistan: A systematic review
Source: PLoS One. 2018 Jun 21;13(6):e0199413. doi: 10.1371/journal.pone.0199413 (PMC6013248; doi:10.1371/journal.pone.0199413)
Supplement: S1 Table — Outcome of the quality assessment of studies identified from the literature search and meeting the eligibility criteria, using the Newcastle-Ottawa Scale. (DOCX) [file pone.0199413.s007.docx]

|  | **Selection** | | | | Comparability | **Outcome** | |
| --- | --- | --- | --- | --- | --- | --- | --- |
| **Author (Year)** | **Representative-ness of sample** | **Sample size (n)** | **Non-respondents** | **Ascertainment of exposure** | **Comparability based on design and analysis** | **Assessment of outcome** | **Statistical test?** |
| Ahmed et al. (2009)^20^ |  | ***** |  | ***** | ****** | ***** |  |
| Arif et al. (1998)^21^ | ***** | ***** |  | ***** | ****** | ****** |  |
| Fatima et al. (2014)^22^ | ***** |  | ***** | ***** | ****** | ****** |  |
| Hussain et al. (2005)^23^ | ***** | ***** |  | ***** | ****** | ****** |  |
| Khan et al. (2003)^24^ | ***** | ***** | ***** | ***** | ****** | ***** |  |
| Khan et al. (2005)^25^ | ***** | ***** |  | ***** | ****** | ***** |  |
| Khan et al. (2007)^26^ |  |  |  | ***** | ****** | ***** |  |
| Khan & Hussain (2003)^27^ | ***** | ***** |  | ***** | ****** | ****** |  |
| Marsh et al. (1996)^28^ | ***** |  |  | ***** | ****** | ***** | ***** |
| Rizvi & Hussain (2001)^29^ | ***** |  |  | ***** | ****** | ***** |  |
| Shah et al. (2003)^30^ | ***** | ***** |  | ***** | ****** | ***** | ***** |
| Shehzadi et al. (2005)^31^ |  |  |  | ***** | ****** | ****** |  |
