# Supplementary material for: Management of tuberculosis by healthcare practitioners in Pakistan: A systematic review
Source: PLoS One. 2018 Jun 21;13(6):e0199413. doi: 10.1371/journal.pone.0199413 (PMC6013248; doi:10.1371/journal.pone.0199413)
Supplement: S1 Other Outcome Measures — (DOCX) [file pone.0199413.s008.docx]

**Other Outcome Measures**

Non-ISTC Outcomes

Knowledge of the range of symptoms of tuberculosis

While ISTC Standard 1 highlighted the importance of recognising an unexplained cough as the main symptom of tuberculosis, the international protocol did not allude to the range of symptoms that a tuberculosis patient may present with - despite it being an important aspect of diagnosis. Pakistan’s national guidelines do, however, acknowledge a diversity of TB symptoms, which can include weight loss, chest pain, loss of appetite, fever, night sweats and fatigue. Three studies provided data on this, showing knowledge of symptoms other than a cough was inadequate. With a moderate degree of heterogeneity, the percentage of practitioners who recognised that symptoms may vary between patients ranged from 12.5% [24] to 45% [30] , as can be observed in **S3 Fig**.

Diagnosing and treating patients personally instead of referring elsewhere

Although the ISTC does not specifically advise the referral of patients to specialist practitioners, except in named circumstances such as repeated treatment failure, multi-drug resistant disease or for treatment of co-morbidities, there has been a nationwide effort to encourage public-private partnerships across Pakistan; there is some reporting on the referral behaviours of practitioners. Five studies provide such data. Between 17.5% [22] and 80% [30] of doctors diagnosed a suspected TB patient themselves, instead of referring them to another doctor. Additionally, between 29.1% [22] and 83% [30] of practitioners treated a (diagnosed) TB patient themselves, instead of referring elsewhere. Heterogeneity was seen in both outcome measures (**S4 Fig** and **S5 Fig)**, though the former outcome was slightly more heterogeneous than the latter. While four of the five studies showed that up to around 70% of the practitioners diagnosed TB patients personally, these same studies showed that nearly 60% treated patients themselves, which implies a moderate, though limited, level of patient referral.
